# Relationships Between Omega-3 Index and Cardiometabolic Disease Risk Factors in Collegiate Athletes

**DOI:** 10.3390/nu18182974

**Published:** 2026-09-11

**Authors:** Andrew R. Jagim, Ward Dobbs, Ben Skramstad, Ava Kloehn, Taylor Molling, Kari Johnson, Jennifer B. Fields, Margaret T. Jones

**Affiliations:** 1 Health Professions Department, University of Wisconsin—La Crosse, La Crosse, WI 54601, USA; skramstad1091@uwlax.edu; 2 Division of Kinesiology and Health, University of Wyoming, Laramie, WY 82071, USA; wdobbs@uwyo.edu; 3 Biology Department, University of Wisconsin—La Crosse, La Crosse, WI 54601, USA; kloehn8017@uwlax.edu (A.K.); molling1318@uwlax.edu (T.M.); kjohnson4@uwlax.edu (K.J.); 4 Department of Nutritional Sciences, University of Connecticut, Storrs, CT 06268, USA; jennifer.fields@uconn.edu; 5 Kinesiology, Sport, and Hospitality Management, George Mason University, Fairfax, VA 22030-4444, USA; mjones15@gmu.edu

**Keywords:** Omega-3, essential fatty acids, cardiovascular disease, lipids, athlete health

## Abstract

**Background/Objectives**: The omega-3 index, defined as the percentage of eicosapentaenoic acid (EPA) and docosahexaenoic acid (DHA) in erythrocyte membranes, is an established biomarker of long-term omega-3 fatty acid status and cardiometabolic disease risk in clinical populations. The primary purpose of this cross-sectional study was to determine the prevalence of National Collegiate Athletic Association Division III men’s and women’s collegiate athletes with an Omega-3 Index below the recommended threshold of 4%. **Methods**: Fifty (male, *n* = 21; female, *n* = 29) collegiate athletes completed a single testing session including anthropometrics, body composition, resting heart rate (HR) and blood pressure assessment, and venous blood sampling for determination of Omega-3 Index, complete blood counts, and lipid profiles. The cardiovascular risk factors examined were total cholesterol, HDL, LDL, body mass index, body composition, waist-to-hip ratio, resting hemodynamics, and blood glucose. **Results**: Overall, 54% of athletes exhibited an Omega-3 Index < 4%, while 100% had an Omega-3 Index < 8%. Male athletes demonstrated significantly higher Omega-3 Index, total omega-3 fatty acid levels, and lower omega-6:omega-3 ratios than females (*p* ≤ 0.005; ηp^2^ = 0.154–0.194). Pearson correlation analyses revealed few significant correlations between Omega-3 Index and cardiometabolic disease risk factors, with only weak relationships observed for systolic blood pressure (r = 0.284, *p* = 0.046) and triglycerides (r = −0.348, *p* = 0.035). **Conclusions**: Suboptimal omega-3 status was highly prevalent among collegiate athletes, with no participant achieving the cardioprotective Omega-3 Index threshold of ≥8%. Although male athletes demonstrated a more favorable omega-3 fatty acid profile than females, Omega-3 Index exhibited limited correlations with traditional cardiometabolic disease risk factors in this young, healthy population.

## 1. Introduction

It is well known that dietary habits and the intake of specific nutrients can have a profound impact on the health and performance capabilities of athletes [1]. Athletes are often encouraged to eat a well-balanced diet with a mixture of lean protein, healthy fats, and fruits and vegetables for daily fiber and micronutrient intake. Dietary fat intake has been shown to exert varying effects pertaining to risk factors for disease, depending on the type of fat being consumed and corresponding nutrient profiles and chemical composition. The Omega-3 polyunsaturated fatty acids eicosapentaenoic acid (EPA; 20:5) and docosahexaenoic acid (DHA; 22:6) are essential fat-derived nutrients that must be obtained through the diet [2,3]. These fatty acids exhibit an array of biological functions and have been shown to confer multiple cardiovascular and neurological health benefits and play critical roles in cardiovascular function, inflammatory regulation, and neuronal membrane integrity [2,3,4].

While there is speculation regarding the precise role of Omega-3 fatty acids in cardiovascular disease [5], the evidence to date suggests that EPA and DHA intake effectively reduce the incidence of certain cardiovascular-related disease outcomes in a dose-dependent manner [6,7]. For example, EPA is typically recognized as the Omega-3 fatty acid component responsible for positive outcomes [3]; however, DHA is also associated with improvement in risk factors for cardiovascular disease, particularly those known to affect some collegiate student athletes, namely, hypertension [8] and hyperlipidemia [9,10]. The Omega-3 fatty acids EPA and DHA are thought to have independent and shared, complementary and synergistic neuro- and cardio-protective effects and might aid heart and brain health in athletes to a greater extent when combined [11]. However, less is known about the potential sex differences and how they may subsequently influence these cardiovascular benefits, particularly among an athletic population.

The Omega-3 index represents the sum of EPA and DHA levels in erythrocyte membranes, expressed as a percentage of total erythrocyte fatty acids [12,13], and serves as a clinically relevant biomarker of cardiovascular disease risk. An Omega-3 Index ≥ 8% is considered cardioprotective, whereas values <4% are associated with elevated cardiometabolic risk in general populations [12,14], with current research supporting an inverse relationship with cardiovascular disease risk and Omega-3 Index [14]. Although Omega-3 fatty acids can be obtained through the diet, many individuals, including athletes [15,16], consume very low amounts, with several recent studies confirming that a large percentage of athletes have a low Omega-3 Index [15,16]. Moreover, humans [17], especially males [18], have a limited ability to synthesize EPA and DHA from the plant-based precursor Omega-3 fatty acid, alpha-linolenic acid (18:3). The most common and readily available sources of dietary EPA and DHA are fish, seafood, and supplements (from algae or fish). The challenge for most people is to consume sufficient daily amounts of these Omega-3 fatty acids to benefit from their cardioprotective effects. A recent meta-analysis suggests that 2 g per day of mixed EPA + DHA intake is likely needed to optimize Omega-3 fatty acid profiles [4]. As such, Omega-3 fatty acid supplementation would likely be warranted to provide a cardiovascular benefit if Omega-3 Index values are identified to be low (<4%).

Although athletes are typically characterized as metabolically healthy, emerging evidence suggests that cardiovascular risk factors may still be present in certain athletic populations [8,9,10]. Furthermore, omega-3 fatty acids have been implicated in neuromuscular function, exercise recovery, and neuroprotection, thereby providing support for omega-3 fatty acids beyond their cardiovascular-related health benefits for athlete populations. To date, preliminary findings indicate that a low percentage of collegiate athletes have an Omega-3 Index within recommended ranges, with mean Omega-3 Index values ranging from 4.4 to 4.6% [16,19,20]. Despite the physiological relevance of omega-3 fatty acids for both cardiovascular and neurological health, limited data exist describing Omega-3 Index status in collegiate athletes, with even fewer studies available examining sex-specific differences in Omega-3 Index profiles. Additionally, the relationship between Omega-3 Index and markers of cardiometabolic health has not been well characterized in a population of Division III collegiate athletes; a population that may be at particular risk because of the lack of financial resources available to them. Therefore, the primary purpose of this study was to examine the prevalence of collegiate athletes with an Omega-3 Index below 4%. A secondary aim was to explore potential sex differences in Omega-3 Index status and to examine relationships between Omega-3 Index and cardiometabolic disease risk factors.

## 2. Materials and Methods

### 2.1. Study Design

This investigation employed a cross-sectional design to assess omega-3 status and its associations with cardiovascular risk factors in collegiate athletes. All data were collected during a single laboratory visit during which participants were assessed for height, weight, body composition, heart rate, blood pressure, and waist circumference measurements. Participants then completed a blood draw which was later assessed for whole blood Omega-3 fatty acid levels, lipid profiles, and a complete blood count. Prior to testing, participants were asked to refrain from intense physical activity for at least 12 h and refrain from consuming food or energy-containing beverages for at least 8 h.

### 2.2. Study Participants

Fifty National Collegiate Athletics Association (NCAA) Division III collegiate athletes (male, *n* = 21; female, *n* = 29) between 18 and 25 years of age from a single academic institution were recruited from a university athletic program to participate in the current study. Inclusion criteria included active participation in collegiate athletics without restriction and clearance for full sport participation. Participants were excluded if they were: currently injured (including concussive injury), unable to participate in regular training, using long-term anti-inflammatory therapy (≥20 days), using anti-hypertensive medications, and using medications known to alter lipid metabolism. All participants provided written informed consent prior to participation. The study was approved by the University Institutional Review Board (Approved On: 9 October 2025; IRB_26-WD-66).

### 2.3. Study Procedures

#### 2.3.1. Anthropometrics and Body Composition

Height and body mass were measured using a calibrated stadiometer and digital scale, which were used to calculate body mass index (BMI). Body composition was assessed using a multi-frequency bioelectrical impedance analyzer device (H20N scale, InBody Inc., Cerritos, CA, USA). Body composition parameters included body fat percentage (BF%), and fat-free mass (FFM). Waist and hip circumference were measured using a measuring tape with measurements taken at the narrowest and widest sections of the abdomen and buttocks areas, respectively. These values were later expressed as a waist-to-hip ratio (WHR).

#### 2.3.2. Resting Hemodynamic Assessment

Resting heart rate and blood pressure were measured following 5 min of seated rest using an automated sphygmomanometer (Riester USA LLC, Chapel Hill, NC, USA). Three readings were obtained and averaged. Resting heart rate and blood pressure were assessed using standard clinical assessment guidelines. Systolic and diastolic blood pressure values were used as part of the determination of risk factors for cardiovascular disease.

#### 2.3.3. Blood Collection and Laboratory Analyses

A venous blood sample was obtained by trained personnel. Fasted supine blood samples were collected via venipuncture from the antecubital fossa region using standard phlebotomy procedures. Samples were collected in spray-coated K2 ethylenediaminetetraacetic acid (EDTA) vacutainer tubes and serum vacutainer tubes with no additive (BD Diagnostics, Franklin Lakes, NJ, USA). Samples were processed for a complete blood count with platelet differentials. A standard lipid profile (total cholesterol, HDL-C, LDL-C, triglycerides), along with blood glucose values were also assessed with samples obtained using a fingerstick and dispensed into a test cassette for analysis. Lipid profiles were determined using a sample of whole blood and analyzed using a CLIA-waived Cholestech LDX™ analyzer (Abbott Core Laboratory Systems, Lake Forest, IL, USA).

Omega-3 Index (EPA + DHA as % of total erythrocyte fatty acids) was determined using a Complete Omega-3 assessment kit (Omegaquandt, Sioux Falls, SD, USA). This test sample was collected via fingerstick, after which a blood smear was collected and contained in a transportable individual kit. Each kit was mailed to the manufacturer for plasma fatty acid composition analysis. The Omega-3 Index Complete Test provided a measure of all the fatty acids in the blood and reported levels of 24 fatty acids as well as the Omega-3 Index, Ratios, and the Trans Fat Index, expressed as a percentage of total fatty acids.

### 2.4. Statistical Analysis

Normality of the data was assessed using visual inspection of histograms and the Shapiro–Wilk test. Homogeneity of variance between groups was evaluated using Levene’s test. Prevalence of Omega-3 Index < 4% and <8%was calculated as a percentage of the total sample. Descriptive statistics are presented as mean ± standard deviation (SD).

Between-sex differences in participant characteristics, lipid profiles, complete blood counts, erythrocyte fatty acid composition and derived fatty acid indices were examined using univariate general linear models, with sex included as the fixed factor. When a significant main effect of sex was identified, pairwise comparisons were performed using the least significant difference (LSD) procedure. Estimated mean differences, 95% confidence intervals (CI), and corresponding *p*-values were reported. Effect sizes for between-sex comparisons were quantified using partial eta squared (ηp^2^), with values of approximately 0.01, 0.06, and 0.14 interpreted as small, medium, and large effects, respectively. Because multiple related outcomes were examined, no formal adjustment for multiple comparisons was applied to the exploratory analyses. Therefore, individual *p*-values, particularly those near the threshold for statistical significance, should be interpreted cautiously and in conjunction with the magnitude of the observed effect, corresponding confidence intervals, and the overall pattern of findings.

Pearson product-moment correlation coefficients (r) were calculated to examine relationships between Omega-3 Index and cardiovascular disease risk factors, including anthropometric characteristics, resting hemodynamics, lipid profile variables, and fasting blood glucose. Correlation coefficients were interpreted using the following criteria: negligible (<0.20), weak (0.20–0.39), moderate (0.40–0.59), strong (0.60–0.79), and very strong (≥0.80). Statistical significance was established a priori at *p* < 0.05. Scatter plots were generated to visually inspect the relationships between Omega-3 Index and each cardiovascular disease risk factor and to assess the presence of potential non-linear patterns or influential observations. Data were analyzed using SPSS V.28 (IBM Corporation, Armonk, NY, USA).

## 3. Results

A total of 50 NCAA Division III collegiate athletes (male, *n* = 21; female, *n* = 29) completed all anthropometric assessments. Participant characteristics are presented in Table 1. Participants from the following men’s sports were included in the analysis: track and field, *n* = 11; football, *n* = 3; and wrestling, *n* = 7. Participants from the following women’s sports were included in the analysis: track and field, *n* = 20; soccer, *n* = 4; volleyball, *n* = 3; and cross country, *n* = 2. There was no difference in age between male and female athletes (19.6 ± 1.3 vs. 19.6 ± 1.2 years, *p* = 0.956). Male athletes were significantly taller (181.8 ± 7.8 vs. 165.6 ± 8.7 cm, *p* < 0.001) and heavier (81.9 ± 15.8 vs. 64.4 ± 9.9 kg, *p* < 0.001) than female athletes. Although BMI did not differ between sexes (*p* = 0.065), males exhibited significantly lower BF% (12.4 ± 5.7% vs. 20.5 ± 4.8%, *p* < 0.001), greater FFM (71.3 ± 11.6 vs. 51.0 ± 6.4 kg, *p* < 0.001), higher fat-free mass index (21.5 ± 2.6 vs. 18.7 ± 2.4 kg·m^−2^, *p* < 0.001), and a greater WHR (0.80 ± 0.00 vs. 0.70 ± 0.10, *p* < 0.001).

Resting hemodynamic variables are presented in Table 1. Male athletes demonstrated higher systolic blood pressure than females (136.9 ± 10.7 vs. 117.8 ± 10.6 mmHg, *p* < 0.001) as well as higher diastolic blood pressure (73.1 ± 6.9 vs. 68.4 ± 6.1 mmHg, *p* = 0.040). Conversely, resting heart rate was lower in males compared with females (58.4 ± 12.5 vs. 69.3 ± 13.4 beats·min^−1^, *p* = 0.014).

Frequency analysis demonstrated that 27 of the 50 athletes (54.0%) exhibited an Omega-3 Index below the clinically relevant threshold of 4%. The prevalence of Omega-3 Index values below 4% was greater among female athletes (18/29; 62.1%) than male athletes (9/21; 42.9%); however, this difference was not statistically significant (χ^2^(1) = 1.81, *p* = 0.18). Furthermore, all participants (100%) had an Omega-3 Index below the cardioprotective threshold of 8%, with no differences observed between sexes. Across the entire cohort, the mean Omega-3 Index was 4.10 ± 0.85%, the Omega-6:Omega-3 ratio was 10.80 ± 2.27, and total omega-3 fatty acid level was 3.91 ± 0.88%.

**Figure 1:**
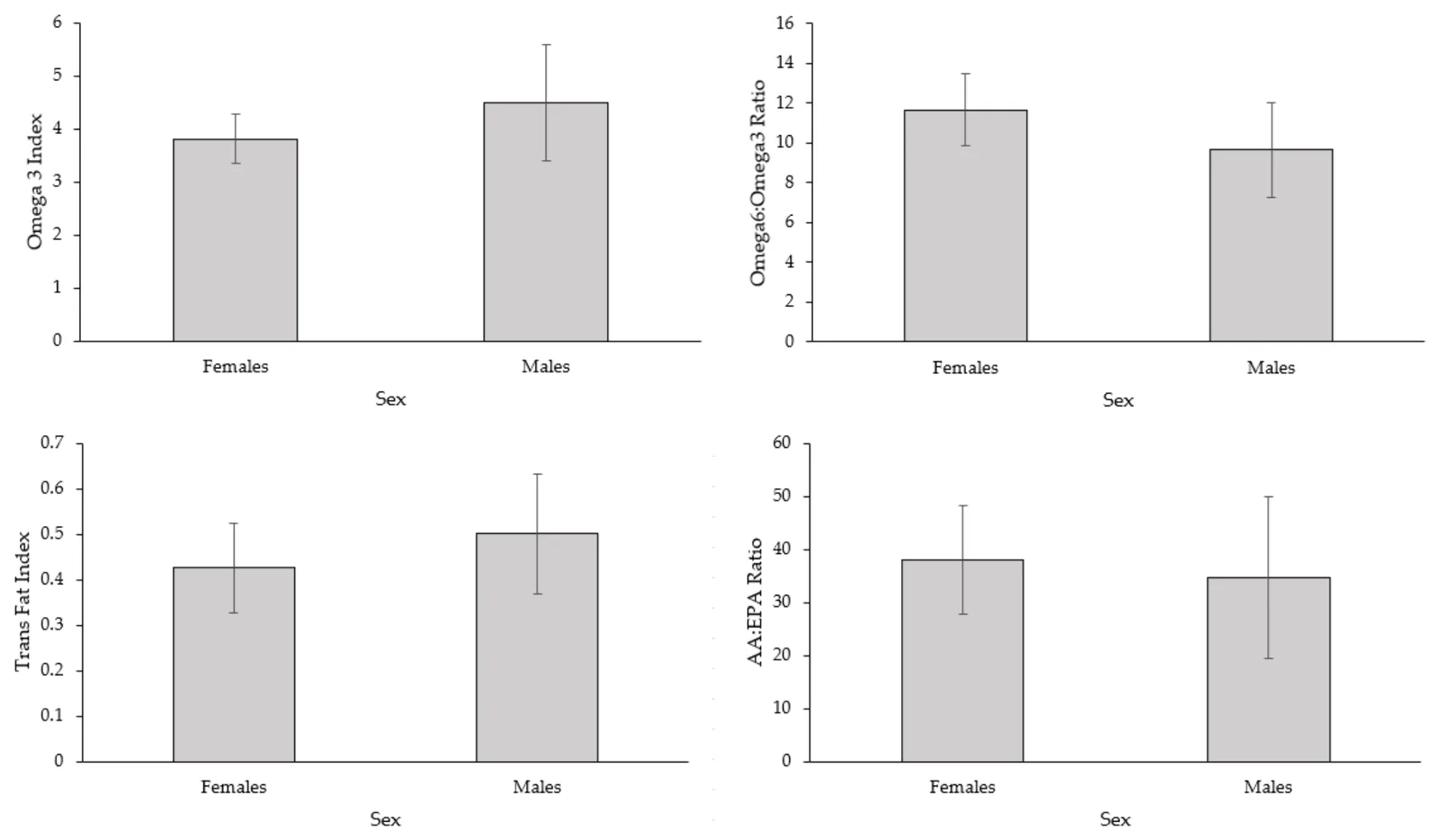
Sex Differences in Erythrocyte Fatty Acid Biomarkers in Collegiate Athletes.

Significant between-sex differences were observed for several fatty acid biomarkers (Figure 1). Male athletes demonstrated a higher Omega-3 Index than female athletes (4.49 ± 1.09% vs. 3.81 ± 0.46%, respectively), F(1, 48) = 9.19, *p* = 0.004, ηp^2^ = 0.161. Pairwise comparisons indicated a mean difference of 0.684% (95% CI: 0.230 to 1.137%). Total omega-3 fatty acid levels were significantly greater in males than females (4.32 ± 1.13% vs. 3.62 ± 0.49%), F(1, 48) = 8.74, *p* = 0.005, ηp^2^ = 0.154, corresponding to a mean difference of 0.696% (95% CI: 0.223 to 1.169%).

Males also exhibited a lower Omega-6:Omega-3 ratio than females (9.64 ± 2.36 vs. 11.64 ± 1.82), F(1, 48) = 11.54, *p* = 0.001, ηp^2^ = 0.194. Pairwise comparisons demonstrated a mean difference of −2.00 (95% CI: −3.19 to −0.82). Likewise, total omega-6 fatty acid level was lower in males than females (39.29 ± 2.55% vs. 41.34 ± 1.75%), F(1, 48) = 11.35, *p* = 0.001, ηp^2^ = 0.191, with a mean difference of −2.05% (95% CI: −3.27 to −0.83). Additional between-sex differences were identified for total saturated fatty acids, which were significantly higher in males than females (35.84 ± 2.12% vs. 34.43 ± 1.33%), F(1, 48) = 8.36, *p* = 0.006, ηp^2^ = 0.148. Males also demonstrated greater Trans Fat Index values (0.501 ± 0.132 vs. 0.426 ± 0.098), F(1, 48) = 5.42, *p* = 0.024, ηp^2^ = 0.101, as well as higher total trans fatty acid levels (0.595 ± 0.154% vs. 0.509 ± 0.110%), F(1, 48) = 5.36, *p* = 0.025, ηp^2^ = 0.100.

No statistically significant between-sex differences were observed for the AA:EPA ratio (F(1, 48) = 0.91, *p* = 0.345, ηp^2^ = 0.019) or total monounsaturated fatty acid levels (F(1, 48) = 0.09, *p* = 0.770, ηp^2^ = 0.002). Overall, the largest between-sex differences were observed for the Omega-6:Omega-3 ratio (ηp^2^ = 0.194), total omega-6 fatty acids (ηp^2^ = 0.191), and Omega-3 Index (ηp^2^ = 0.161), with moderate effect sizes also evident for total omega-3 fatty acids (ηp^2^ = 0.154) and total saturated fatty acids (ηp^2^ = 0.148). Smaller but statistically significant effects were observed for the Trans Fat Index (ηp^2^ = 0.101) and total trans fatty acids (ηp^2^ = 0.100).

**Figure 2:**
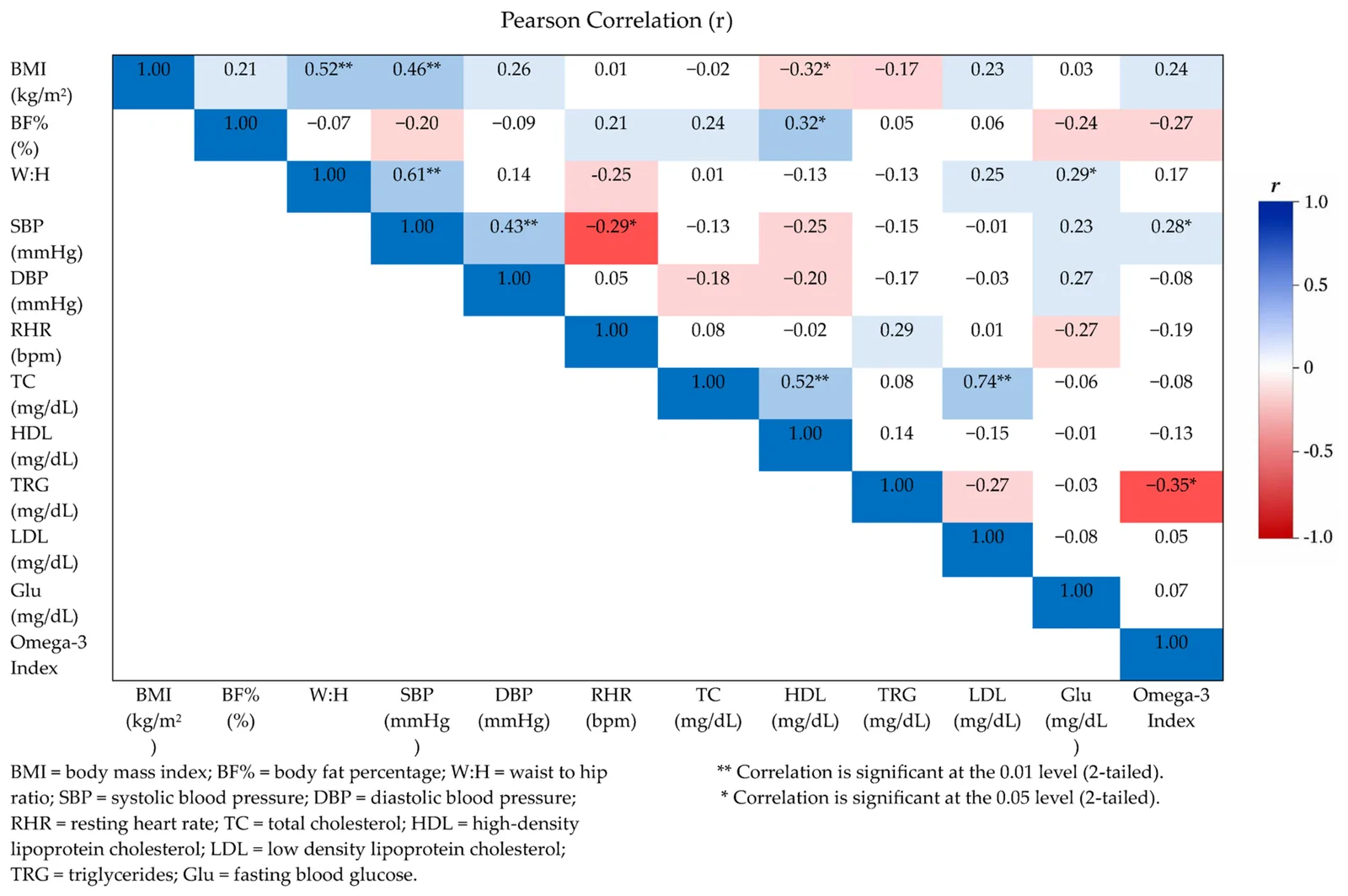
Relationships between Omega-3 Index and risk factors associated with cardiovascular disease.

Pearson correlation coefficients examining the relationships between Omega-3 Index, anthropometric characteristics, resting hemodynamics, and cardiometabolic biomarkers are presented in Figure 2. Overall, Omega-3 Index demonstrated few significant correlations with the measured cardiovascular disease risk factors. A weak positive correlation was observed between Omega-3 Index and systolic blood pressure (r = 0.284, *p* = 0.046), whereas a weak inverse correlation was identified between Omega-3 Index and triglyceride levels (r = −0.348, *p* = 0.035). No significant relationships were observed between Omega-3 Index and body mass index (BMI; r = 0.244, *p* = 0.087), body fat percentage (r = −0.271, *p* = 0.057), waist-to-hip ratio (r = 0.171, *p* = 0.236), diastolic blood pressure (r = −0.082, *p* = 0.593), resting heart rate (r = −0.191, *p* = 0.173), total cholesterol (r = −0.082, *p* = 0.580), HDL cholesterol (r = −0.131, *p* = 0.371), LDL cholesterol (r = 0.045, *p* = 0.795), or fasting blood glucose (r = 0.066, *p* = 0.651). 

Several significant correlations were observed among the cardiovascular risk factors themselves. BMI demonstrated a moderate positive correlation with waist-to-hip ratio (r = 0.519, *p* < 0.001) and systolic blood pressure (r = 0.458, *p* < 0.001), along with a weak inverse correlation with HDL cholesterol (r = −0.318, *p* = 0.026). Waist-to-hip ratio was positively correlated with systolic blood pressure (r = 0.612, *p* < 0.001) and weakly correlated with fasting blood glucose (r = 0.291, *p* = 0.041). Systolic blood pressure demonstrated a moderate positive correlation with diastolic blood pressure (r = 0.433, *p* = 0.003) and a weak inverse correlation with resting heart rate (r = −0.292, *p* = 0.039).

Body fat percentage was positively correlated with HDL cholesterol (r = 0.317, *p* = 0.026). Total cholesterol demonstrated a strong positive correlation with LDL cholesterol (r = 0.741, *p* < 0.001) and a moderate positive correlation with HDL cholesterol (r = 0.518, *p* < 0.001). No additional statistically significant correlations were observed among the remaining variables. Visual inspection of scatter plots did not reveal consistent evidence of non-linear relationships between Omega-3 Index and the cardiovascular disease risk factors examined. The observed graphical patterns were generally consistent with the Pearson correlation analyses, with most relationships appearing weak or negligible. Scatter plots depicting relationships between Omega-3 Index and cardiometabolic disease risk factors can be found in Supplementary Materials (Figure S1).

Clinical chemistry and lipid profile variables are summarized in Table 2. Total cholesterol, triglycerides, LDL cholesterol, non-HDL cholesterol, and the LDL:HDL ratio did not differ between male and female athletes (*p* > 0.05 for all comparisons). Female athletes demonstrated higher HDL cholesterol than males (64.5 ± 17.2 vs. 53.9 ± 12.1 mg·dL^−1^, *p* = 0.046), whereas males exhibited greater fasting blood glucose levels than females (89.6 ± 6.7 vs. 83.0 ± 7.5 mg·dL^−1^, *p* = 0.008).

## 4. Discussion

The primary purpose of this study was to characterize omega-3 status in a cohort of collegiate athletes by determining the prevalence of individuals with an Omega-3 Index below the clinically relevant threshold of 4%. A secondary objective was to examine potential sex differences in the Omega-3 Index and explore relationships between the Omega-3 Index and traditional CVD risk factors. The principal findings were that approximately one-half of the athletes demonstrated an Omega-3 Index below 4%, suggesting that inadequate omega-3 status remains common even among healthy collegiate athletes. Moreover, sex explained a large proportion of the variance in Omega-3 Index, Omega-6:Omega-3 ratio, total omega-6 fatty acids, total omega-3 fatty acids, and total saturated fatty acids (ηp^2^ = 0.148–0.194), whereas differences in Trans Fat Index and total trans fatty acids were associated with medium effect sizes (ηp^2^ ≈ 0.10). Third, despite these sex differences in fatty acid composition, Omega-3 Index demonstrated few significant relationships with traditional cardiovascular risk factors, with only weak correlations observed for systolic blood pressure and triglycerides. Finally, several expected relationships among anthropometric variables, blood pressure, and lipid biomarkers were observed, supporting the internal consistency of the dataset.

The finding that approximately 50% of participants exhibited an Omega-3 Index below 4% is consistent with previous investigations demonstrating inadequate Omega-3 status among active young adults [16,20,21,22]. Anzalone et al. [16] similarly observed mean Omega-3 Index near 4.4% in Division I football players. Likewise, Heileson and colleagues demonstrated average Omega-3 Index values between 4.4 and 4.6% in collegiate athletes from various sports [20]. Together, these studies indicate that despite generally favorable health behaviors and elevated physical activity levels, many collegiate athletes fail to consume sufficient EPA and DHA to achieve levels considered cardioprotective. The present findings extend this growing body of literature by demonstrating that inadequate omega-3 status also exists among this cohort of Division III athletes, a population that has received considerably less scientific attention than Division I athletes. Moreover, Division III institutions typically have fewer nutritional resources available to student athletes, including limited access to sports dietitians, individualized nutrition counseling, and structured supplementation programs. Consequently, athletes competing at this level may be at even greater risk of inadequate dietary omega-3 intake. Although dietary intake was not assessed in the present investigation, the observed Omega-3 Index likely reflect insufficient habitual consumption of fatty fish and other EPA- and DHA-rich foods, consistent with previous reports among collegiate athletes.

Although an Omega-3 Index below 4% has consistently been associated with increased cardiovascular risk in middle-aged and older adults [3,12], the clinical significance of this threshold in collegiate athletes remains uncertain. Young athletes typically exhibit favorable lipid profiles, lower adiposity, greater cardiorespiratory fitness, and lower overall cardiovascular risk compared with the general population [23,24,25,26]. Consequently, the absence of significant relationships between Omega-3 Index and cardiovascular disease risk factors observed in the current study is perhaps unsurprising. Overall, the study cohort demonstrated generally favorable anthropometric and cardiometabolic health characteristics. Expected sex-related differences were observed for body composition, blood pressure, resting heart rate, HDL cholesterol, and fasting glucose, while most traditional lipid profile variables were comparable between male and female athletes. The relatively narrow range of blood pressure, glucose, lipid levels, and body composition variables likely reduced the ability to detect meaningful correlations. Furthermore, the protective effects of habitual exercise [25] may attenuate the influence of omega-3 status on conventional cardiovascular biomarkers, particularly in young adults without overt metabolic dysfunction.

Despite the high prevalence of inadequate omega-3 status among this cohort, Omega-3 Index demonstrated only weak correlations with traditional cardiometabolic disease risk factors. Significant correlations were limited to a weak positive relationship with systolic blood pressure and a weak inverse relationship with triglyceride levels, while no significant correlations were observed with BMI, body fat percentage, waist-to-hip ratio, resting heart rate, total cholesterol, HDL cholesterol, LDL cholesterol, fasting glucose, or diastolic blood pressure. These findings differ somewhat from studies conducted in older or clinical populations in which higher omega-3 status has been associated with improved blood pressure regulation, and reduced cardiovascular mortality [2,3,12,14]. However, these discrepancies are likely attributable to differences in participant characteristics rather than conflicting physiological mechanisms. Athletes generally demonstrate limited variability in these biomarkers [25], as was also observed in the current study, which introduces a potential floor effect whereby improvements associated with higher omega-3 status may become difficult to detect. Consequently, the lack of significant correlations observed in the present study should not necessarily be interpreted as evidence that omega-3 fatty acids lack physiological importance in athletic populations.

A novel finding of the present study was the observation that male athletes demonstrated significantly greater Omega-3 Index and total omega-3 fatty acid levels than female athletes, accompanied by lower omega-6:omega-3 ratios and lower total omega-6 fatty acid levels. These differences were associated with moderate-to-large effect sizes (ηp^2^ = 0.15–0.19), suggesting that biological sex explained a meaningful proportion of the variability in erythrocyte fatty acid composition. Previous work has suggested that biological sex may influence omega-3 metabolism, with females often exhibiting greater endogenous conversion of alpha-linolenic acid to DHA, potentially mediated through estrogen-dependent regulation of desaturase enzymes [27]. While this metabolic advantage has been demonstrated under controlled dietary conditions, erythrocyte Omega-3 Index ultimately reflects long-term intake of pre-formed EPA and DHA rather than endogenous synthesis alone. Consequently, the higher Omega-3 Index observed in males within the present study may reflect behavioral rather than physiological differences, including greater seafood consumption, increased supplement use, or sport-specific dietary practices [28,29]. Differences in fish and seafood consumption, use of omega-3-containing supplements, and other dietary behaviors may have contributed to the higher Omega-3 Index observed among male athletes in the present study [28,29]. In addition, sport type may represent an important source of variability as athletes participating in different sports may have distinct nutritional demands, body composition goals, and dietary practices, each of which could influence habitual intake of EPA and DHA or the use of omega-3 supplements. However, because dietary omega-3 intake, fish and seafood consumption, supplement use, and sport-specific nutritional practices were not assessed in the present investigation, the relative contribution of these behavioral factors cannot be determined. Therefore, the observed sex differences should be interpreted cautiously and should not be attributed solely to sex-specific physiological differences in omega-3 metabolism. Future studies should incorporate detailed dietary assessment, omega-3 supplementation history, and adequately powered sport-specific analyses to better characterize the behavioral and physiological determinants of Omega-3 Index among male and female athletes.

From a practical perspective, the current findings reinforce recommendations encouraging athletes to consume adequate dietary sources of EPA and DHA or consider supplementation when habitual intake is insufficient. Current International Society of Sports Nutrition recommendations suggest approximately 2 g·day^−1^ of combined EPA and DHA may be necessary to optimize omega-3 status in many individuals, although requirements likely vary according to body size, baseline omega-3 status, and dietary intake [3]. Given that one-half of the present cohort exhibited Omega-3 Index values below the 4% threshold, routine assessment of omega-3 status may help identify athletes who could benefit from targeted nutritional interventions. However, whether increasing Omega-3 Index translates into measurable improvements in cardiovascular health or athletic performance among otherwise healthy athletes remains to be determined.

Several limitations should be acknowledged. First, the cross-sectional design precludes any conclusions regarding causality between Omega-3 Index and cardiovascular risk factors. Second, the relatively modest sample size may have limited statistical power to detect small correlations, particularly following stratification by sex, and reduces the precision of the estimated prevalence of Omega-3 Index values below clinically relevant thresholds. Accordingly, the proportion of athletes in the present study with an Omega-3 Index < 4% should be interpreted as a cohort-specific estimate rather than a definitive prevalence estimate for the broader collegiate athlete population. Third, participants were recruited from a single NCAA Division III institution, potentially limiting generalizability to athletes competing at other institutions, competitive levels, geographic regions, and sports. Differences in dietary patterns, seafood consumption, supplement use, sex distribution, sport-specific demands, and access to sports nutrition resources may influence omega-3 status across collegiate athletic populations. Larger multisite investigations with more diverse representation across NCAA divisions, sports, sexes, and geographic regions are needed to establish more precise population-level estimates of omega-3 status among collegiate athletes. Fourth, dietary omega-3 intake, fish consumption, and supplement use were not quantified, preventing evaluation of factors contributing to individual differences in Omega-3 Index.

## 5. Conclusions

In this cohort of NCAA Division III collegiate athletes, approximately one-half of participants demonstrated an Omega-3 Index below the clinically recognized threshold of 4%, indicating that inadequate omega-3 status remains common despite participation in competitive athletics. However, Omega-3 Index was not correlated with traditional cardiometabolic disease risk factors in this young, healthy cohort, suggesting that conventional clinical biomarkers may be relatively insensitive to differences in omega-3 status during early adulthood. Future longitudinal and intervention studies incorporating dietary assessment, sport-specific analyses, inflammatory biomarkers, and neurological outcomes are warranted to determine whether improving omega-3 status confers clinically meaningful benefits for athlete health, recovery, and long-term cardiometabolic risk.

## Figures and Tables

**Table 1:** Summary of participant characteristics.

	Sex	*n*	Mean ± SD	95% CI	95% CI	*p* Value
Age (yrs)	M	21	19.6 ± 1.3	18.8	20.1	0.956
	F	29	19.6 ± 1.2	19.1	20.0	
	Total	50	19.5 ± 1.2	19.2	19.9	
Height (cm)	M	21	181.8 ± 7.8	177.3	186.3	<0.001
	F	29	165.6 ± 8.7	162.3	168.9	
	Total	50	170.9 ± 11.3	167.4	174.4	
Weight (kg)	M	21	81.9 ± 15.8	72.8	91.0	<0.001
	F	29	64.4 ± 9.9	60.7	68.2	
	Total	50	70.1 ± 14.5	65.7	74.6	
BMI (kg·m^−2^)	M	21	24.7 ± 3.8	22.5	26.9	0.065
	F	29	23.5 ± 3.4	22.2	24.8	
	Total	50	23.9 ± 3.5	22.8	25.0	
BF%	M	21	12.4 ± 5.7	9.1	15.7	<0.001
	F	29	20.5 ± 4.8	18.7	22.3	
	Total	50	17.9 ± 6.3	15.9	19.8	
FFM (kg)	M	21	71.3 ± 11.6	64.6	78.0	<0.001
	F	29	51.0 ± 6.4	48.6	53.5	
	Total	50	57.6 ± 12.7	53.7	61.6	
FFMI (kg·m^−2^)	M	21	21.5 ± 2.6	20.0	23.0	<0.001
	F	29	18.7 ± 2.4	17.7	19.6	
	Total	50	19.6 ± 2.8	18.7	20.5	
W:H	M	21	0.8 ± 0.0	0.7	0.8	<0.001
	F	29	0.7 ± 0.1	0.7	0.7	
	Total	50	0.7 ± 0.1	0.7	0.7	
SBP (mmHg)	M	21	136.9 ± 10.7	130.7	143.1	<0.001
	F	29	117.8 ± 10.6	113.8	121.9	
	Total	50	124.0 ± 13.8	119.8	128.3	
DBP (mmHg)	M	21	73.1 ± 6.9	68.7	77.4	0.04
	F	29	68.4 ± 6.1	65.9	70.9	
	Total	50	69.9 ± 6.6	67.7	72.1	
RHR (bpm)	M	21	58.4 ± 12.5	51.1	65.6	0.014
	F	29	69.3 ± 13.4	64.2	74.4	
	Total	50	65.7 ± 13.9	61.4	70.0	

BMI = body mass index; BF% = body fat percentage; FFM = fat-free mass; FFMI = fat-free mass index; kg = kilograms; cm = centimeters; m = meters; W:H = waist-to-hip ratio; SBP = systolic blood pressure; DBP = diastolic blood pressure; RHR = resting heart rate; bpm = beats per minute; SD = standard deviation; CI = confidence interval.

**Table 2:** Summary of metabolic panel.

	Sex	*n*	Mean ± SD	95% CI	95% CI	*p* Value
TC	M	21	152.6 ± 23.0	137.9	167.2	0.281
	F	29	162.9 ± 29.0	151.9	173.9	
	Total	50	159.9 ± 27.5	151.2	168.6	
HDL	M	21	53.9 ± 12.1	46.9	60.9	0.046
	F	29	64.5 ± 17.2	57.8	71.1	
	Total	50	60.9 ± 16.4	55.8	66.0	
TRG	M	21	72.0 ± 31.9	50.6	93.4	0.402
	F	29	83.6 ± 38.9	66.3	100.8	
	Total	50	79.7 ± 36.6	66.7	92.7	
LDL	M	21	87.1 ± 26.4	66.8	107.4	0.995
	F	29	87.2 ± 26.8	75.3	99.1	
	Total	50	87.2 ± 26.2	77.5	96.8	
Non-HDL	M	21	97.2 ± 23.7	82.1	112.2	0.72
	F	29	100.3 ± 25.2	90.5	110.0	
	Total	50	99.3 ± 24.5	91.5	107.2	
LDL:HDL ratio	M	21	1.7 ± 0.8	1.1	2.3	0.4
	F	29	1.5 ± 0.7	1.2	1.8	
	Total	50	1.6 ± 0.7	1.3	1.8	
Glu	M	21	89.6 ± 6.7	85.7	93.4	0.008
	F	29	83.0 ± 7.5	80.1	85.9	
	Total	50	85.2 ± 7.8	82.7	87.6	

TC = total cholesterol; HDL = high-density lipoprotein cholesterol; LDL = low-density lipoprotein cholesterol; TRG = triglycerides; Glu = blood glucose; SD = standard deviation; CI = confidence interval.

## Data Availability

The raw data supporting the conclusions of this article will be made available by the authors on request.

## Supplementary Materials

The following supporting information can be downloaded at: https://www.mdpi.com/article/10.3390/nu18182974/s1, Figure S1 Correlations between Omega-3 Index and cardiovascular disease risk factors in collegiate athletes. Scatter plots depict the relationships between Omega-3 Index and (A) body mass index (BMI), (B) body fat percentage, (C) waist-to-hip ratio, (D) systolic blood pressure (SBP), (E) diastolic blood pressure (DBP), (F) resting heart rate (RHR), (G) total cholesterol (TC), (H) high-density lipoprotein cholesterol (HDL), and (I) low-density lipoprotein cholesterol (LDL). Each point represents an individual participant, and solid lines represent the linear regression line. Pearson product-moment correlation coefficients (r), corresponding p-values, and sample sizes are displayed within each panel.
