# Supplementary material for: Relationships Between Omega-3 Index and Cardiometabolic Disease Risk Factors in Collegiate Athletes
Source: Nutrients. 2026 Sep 11;18(18):2974. doi: 10.3390/nu18182974 (PMC13610564; doi:10.3390/nu18182974)
Supplement: Supplementary file 1 [file nutrients-18-02974-s001.zip › nutrients-4503546-supplementary.pdf]

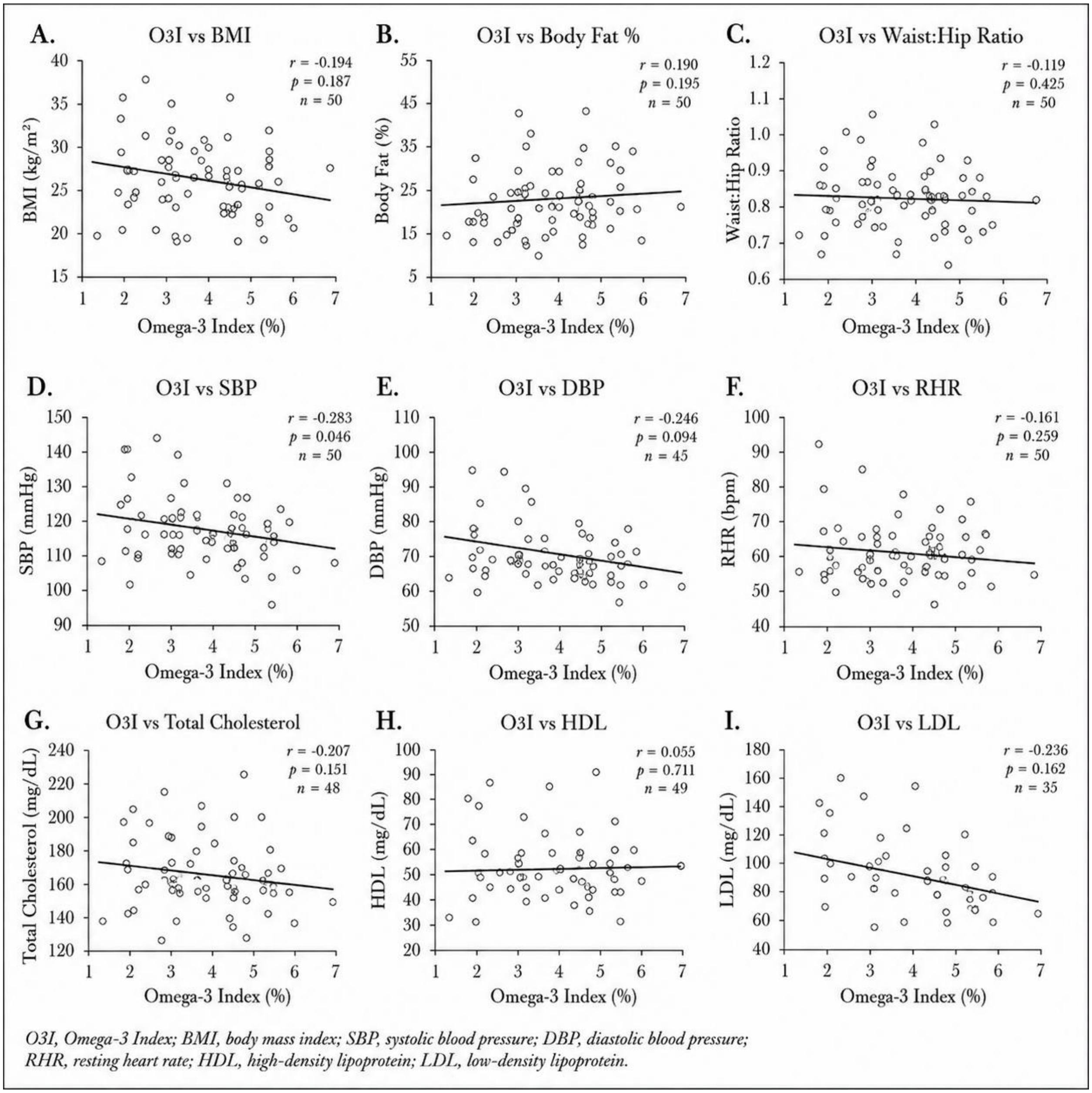

Figure S1. Correlations between Omega-3 Index and cardiovascular disease risk factors in collegiate athletes. Scatter plots depict the relationships between Omega-3 Index and (A) body mass index (BMI), (B) body fat percentage, (C) waist-to-hip ratio, (D) systolic blood pressure (SBP), (E) diastolic blood pressure (DBP), (F) resting heart rate (RHR), (G) total cholesterol (TC), (H) high-density lipoprotein cholesterol (HDL), and (I) low-density lipoprotein cholesterol (LDL). Each point represents an individual participant, and solid lines represent the linear regression line. Pearson product-moment correlation coefficients (r), corresponding p-values, and sample sizes are displayed within each panel.
